# Deep Learning Correction Algorithm for The Active Optics System

**DOI:** 10.3390/s20216403

**Published:** 2020-11-09

**Authors:** Wenxiang Li, Chao Kang, Hengrui Guan, Shen Huang, Jinbiao Zhao, Xiaojun Zhou, Jinpeng Li

**Affiliations:** 1 Nanjing Astronomical Instruments Research Center, University of Science and Technology of China, Hefei 230026, China; lwxiang@mail.ustc.edu.cn (W.L.); kangchao@mail.ustc.edu.cn (C.K.); hengrui@mail.ustc.edu.cn (H.G.); 2 CAS Nanjing Astronomical Instruments Co., Ltd., Nanjing 210042, China; shenghuang@nairc.ac.cn (S.H.); zhaojinbiao@nairc.ac.cn (J.Z.); lijinpeng@nairc.ac.cn (J.L.)

**Keywords:** active optics, wavefront aberrations, deep learning, correction algorithms, heuristic search

## Abstract

The correction of wavefront aberration plays a vital role in active optics. The traditional correction algorithms based on the deformation of the mirror cannot effectively deal with disturbances in the real system. In this study, a new algorithm called deep learning correction algorithm (DLCA) is proposed to compensate for wavefront aberrations and improve the correction capability. The DLCA consists of an actor network and a strategy unit. The actor network is utilized to establish the mapping of active optics systems with disturbances and provide a search basis for the strategy unit, which can increase the search speed; The strategy unit is used to optimize the correction force, which can improve the accuracy of the DLCA. Notably, a heuristic search algorithm is applied to reduce the search time in the strategy unit. The simulation results show that the DLCA can effectively improve correction capability and has good adaptability. Compared with the least square algorithm (LSA), the algorithm we proposed has better performance, indicating that the DLCA is more accurate and can be used in active optics. Moreover, the proposed approach can provide a new idea for further research of active optics.

## 1. Introduction

Active optics, a key technique in large modern telescopes, is applied to correct wavefront aberrations and reduce the influence of the primary mirror’s deformations on the beam quality [1]. Nowadays, active optics technology is widely utilized in the large modern telescope: The 8 m class VLT (Very Large Telescope) [2], the VISTA (Visible and Infrared Telescope for Astronomy) [3], and LSST (Large Synoptic Survey Telescope) [4].

The wavefront aberration of the telescope comes from multiple sources: atmospheric turbulence, manufacturing and assembly errors, and gravity deformation. The atmospheric wavefront aberrations are usually corrected by adaptive optics (AO), while the gravitational wavefront aberrations are corrected by active optics. Active optics detects the wavefront aberrations by the wavefront detection device such as the interferometer, the Shack–Hartmann sensor, and so on. Then the wavefront aberrations are compensated by using actuators at the back of the mirror, which can significantly improve the observation performance of the large telescopes. The active optics can be used not only in telescopes but also in the large-aperture standard mirror for optical detection. The large-aperture standard mirrors using active optics can work in different conditions. Therefore, the method to improve the correction capability of the mirror has a great significance for the development of modern astronomy and optics.

The calculation of the correction force is one of the key points to improve the correction capability of the mirror. Currently, the existing algorithms for active correction can be classified into three parts, including the algorithm based on free-vibration mode [5], bending mode [6], and Zernike mode [7]. The first kind of algorithm is based on free-vibration mode to solve the correction force of each support and the free-vibration surface shape of the mirror, then the correction target can be fitted with the free-vibration surface shape. Finally, the correction force can be obtained. However, it only reflects the intrinsic characteristics of the mirror, which may not be fully realized when the lateral support is included [8]. The second kind of algorithm is based on bending mode. The stiffness matrix of the mirror can be transformed into a series of orthogonal surfaces shape with the same RMS (Root Mean Square), but this approach relies on manual analysis and selection, which reduces the efficiency of correction [9].

The third kind of algorithm is based on Zernike mode to calculate the correction force, in which the least square algorithm is the most widely used [10]. The least square algorithm is efficiently to correct wavefront aberrations based on the response mirror shape of each active support and obtain good performance in actual application. For example, Dai et al. [11] used the least square algorithm to correct the gravitational deformations of a 1.2 m thin mirror and studied the capability of fitting Zernike aberrations. Zhou et al. [12] applied the least square algorithm to fit the correction target, which improving the observation performance of space telescopes. Schwaer et al. [13] utilized the least square algorithm to calculate the correction force to analyze the support system of a lightweight, low-cost telescope. However, the above three types of algorithms strictly depend on the deformation of the mirror, and cannot effectively deal with the various disturbances, such as thermal distortion, and gravity deformation, especially the error in structure and actuator control. These disturbances will bring great influences on the correction capability of the mirror.

The system identification of the neural network can be utilized in the active optics because the neural network can establish the mapping of the systems with disturbances [14,15]. However, the shallow neural network is prone to be affected by the problem of local optimum during training, so the shallow neural network cannot effectively describe systems with disturbances. The development of deep learning in recent years has brought inspiration for solving this problem. The model of the mirror can be replaced by the deep neural network, and the system identification can be transformed into the parameter optimization of the deep neural network. Some studies have noticed the application of deep learning in the field of optics. Guo et al. [16] have used an improved deep convolutional neural network to replace the traditional gradient-based optimization methods, whose idea is to establish the nonlinear mapping between the input point spread functions and the corresponding phase maps of the optical system. Hegde [17] has employed deep learning for optics design optimizations. Using deep learning indirectly to choose initializations and candidate preselection. The big datasets and long-time epochs are no longer needed. Gomez et al. [18] have applied the convolutional neural network to propose a reconstruction algorithm that can correct the image aberrations. Xu et al. [19] have established a control model based on deep learning for adaptive optics systems, the system control model is fitted with a deep neural network. Compared with the traditional control mode, the deep learning control model achieves better performance. All of these applications have brought new enlightenment for active optics. 

In this study, to maintain the good shape of a large-aperture standard mirror under different working conditions, active optics is applied to correct the deformation of the mirror [20]. A deep neural network is developed for a new correction algorithm in the active optics system. This deep learning correction algorithm combines with an actor network and a strategy unit. The actor network aims to fit the mapping of the active optics system with disturbances, minimize the wavefront aberrations, and optimize the correction force. However, it is hard to find the optimal force. The strategy unit is added to optimize the force. The correction force output by the actor network is used as the search basis of the strategy unit, and then corresponding strategies are input into the active optics system to pick out the optimal correction force. Moreover, a heuristic search algorithm is utilized in the strategy unit to further improve the search speed. The proposed integrated algorithm can effectively improve the correction capability to deal with disturbances.

This paper is organized as follows. Section 2 illustrates the used materials and the principle of the deep learning correction algorithm (DLCA) in this study. In Section 3, we study the correction performance of the DLCA and compare the traditional correction algorithm by conducting a series of experiments. Some discussions about the proposed algorithm are given in Section 4. The conclusions are drawn in Section 5.

## 2. Materials and Methods 

### 2.1. Experimental Materials and Data Collection

#### 2.1.1. Simulation Environment

To validate the correction performance of the DLCA, an active optics platform is established. This platform using the finite element analysis software (ANSYS) is based on a 1 m diameter standard mirror. The key components of the standard mirror are shown in Table 1.

**Figure 1:**
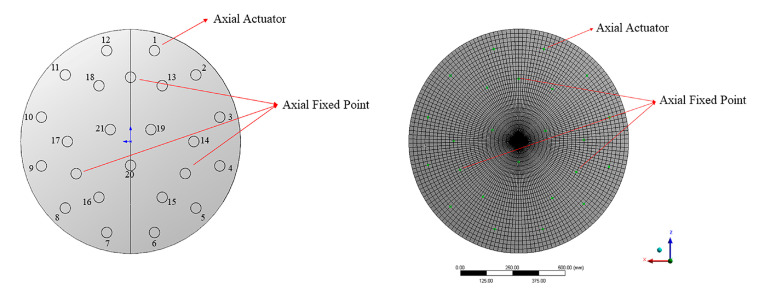
The support system of the standard mirror and finite element analysis.

The support system of the active optics platform is composed of 24 axial supports and 3 lateral supports. The 24 axial supports that include 21 active supports and 3 fixed supports on the back of the mirror, which is distributed on three rings whose radiuses are 121.88 mm (3 supports), 304.86 mm (6 supports), and 444.8 mm (12 support). The fixed supports are distributed on the second support ring to constrain the three degrees of freedom $R_{x}$, $R_{y}$, $R_{z}$ of the mirror. The gravitational deformations of the mirror tend to be more sensitive to the axial supports, so the deformation caused by lateral supports is much less than the axial supports. Therefore, the lateral supports are all composed of passive supports, which are only used to balance the gravity under different working conditions. The support system and finite element analysis (FEA) are shown in Figure 1. The active supports are marked from 1 to 21 in a clockwise direction. The support points are all indicated by red arrows.

#### 2.1.2. Generation of Datasets

In the active optics system, the mirror strictly conforms to Hooke’s law of linearity, which mainly has two aspects:

- The same force always produces the same mirror’s deformation, which is independent of the initial shape of the mirror; that is, the displacement of any point of the mirror is linearly related to the force of the actuator.
- The deformations of the mirror conform to the linear superposition of forces.

Therefore, for the single actuator, the relationship between the deformation of the mirror and the force is linear. The mirror’s deformation can be calculated as

(1) $$W_{i}\left(x,y\right)=F_{i}w_{i}\left(x,y\right)$$

where $W_{i}\left(x,y\right)$ is the deformation of the mirror after $F_{i}$ caused by the i-th actuator, $F_{i}$ is the force exerted by the i-th actuator, $w_{i}\left(x,y\right)$ is the mirror’s deformation caused by the unit force applied by the i-th actuator, which is called the response function of the actuator, and i is the number of actuators.

The matrix composes of each actuator’s response function is called the stiffness matrix of the mirror. The force of each actuator will cause the mirror’s deformation, and the total deformation is the linear superposition of the deformation caused by each actuator. So, the total deformation of the mirror can be expressed as

(2) $$W\left(x,y\right)=\overset{n}{\underset{i=1}{\sum }}W_{i}\left(x,y\right)$$

where W(x,y) is the total deformation of the mirror, $W_{i}\left(x,y\right)$ is the mirror’s deformation caused by the i-th actuator, n is the number of actuators.

Based on the above analysis, the mirror’s deformation and the correction force have the following equation in the active optic system [21,22,23]:

(3) Cf=−W

where C is a stiffness matrix, f is the correction force, W is the mirror’s deformation. When the random force is added, we have the following equation:

(4) C(f+Δf)=−(W+ΔW)

where Δf is the random force, ΔW is the mirror’s deformation caused by Δf. Then, Equation (5) can be obtained by Equations (3) and (4):

(5) CΔf=−ΔW

Based on Equation (5), we can know the relationship between the deformation of the mirror and force, so we can get a large number of data. However, these data do not contain disturbances. To solve this problem, a random disturbance is added to the stiffness matrix to simulate the disturbance of the real system by MATLAB. Therefore, all the training data contain disturbances. In addition, in the real active optics system, it is easier to obtain the training set, because the input and output data already include a variety of disturbances.

### 2.2. Method

#### 2.2.1. The Traditional Correction Algorithm and the DLCA 

**Figure 2:**
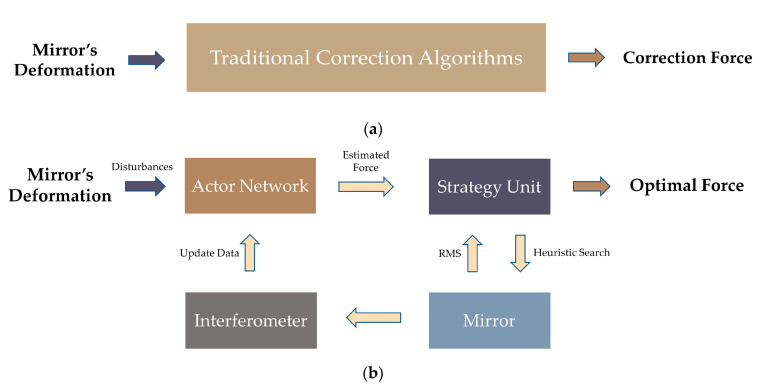
The difference of structure between the traditional correction algorithms and the deep learning correction algorithm (DLCA). (**a**) The structure of the traditional correction algorithm; (**b**) The structure of the DLCA.

In the active optics system, the correction algorithms are aimed at eliminating the wavefront aberrations. However, the traditional correction algorithms directly calculate the correction force based on the deformations of the mirror, as shown in Figure 2a.

In this study, the calculation of the correction force is based on input data and output data. In addition, a strategy unit based on feedback ideas is introduced to optimize the correction force output by the actor network. Then, the possible forces output by the strategy unit will be applied to the mirror, and the RMS of the mirror will be measured by the interferometer, as shown in Figure 2b. In this way, the correction force is not calculated directly, and it will be optimized by the strategy unit, which effectively improves the correction accuracy of the DLCA. In Figure 2b, the actor network and the strategy unit have different roles and the specific contributions are as follows:

- The actor network is developed for quickly outputting the correction force to provide a search basis for the strategy unit, which significantly reduces the search time, and the convergence speed of the DLCA is quickened.
- The strategy unit is used to optimize the correction force output by the actor network to achieve higher correction accuracy.

#### 2.2.2. The Actor Network

**Figure 3:**
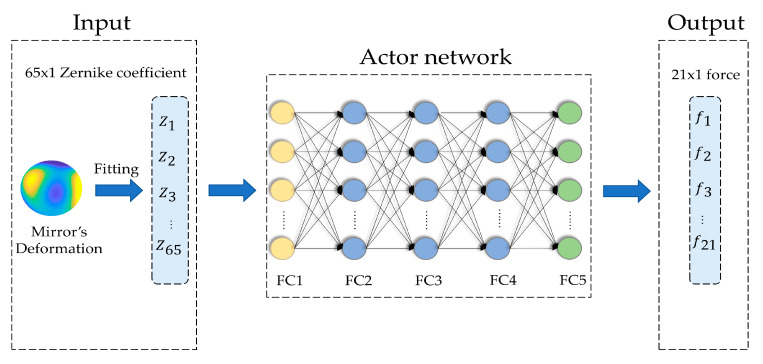
The process of the actor network.

The actor network adopts a deep neural network (DNN) [24]. The DNN can find out complex structures in large datasets by adjusting internal parameters. These parameters use the neuron information of the previous layer to calculate the neuron information of the next layer. Essentially, the DNN can fit any input-to-output mapping with its advantage of automatic feature extraction from data, with no need for any model by manual design [25,26,27]. The DNN is composed of an input layer, multiple hidden layers, and an output layer, which are all fully connected (FC). Starting from the input layer, each layer transforms simple representations into more abstract representations until the output layer. The parameters of the actor network are randomly initialized with small numbers before the training. The process of the actor network is shown in Figure 3, and it includes two main phases: 

##### Forward Propagation Phase

The deformation of the mirror is fitted into 65 Zernike coefficients, which are input to the actor network as a mini-batch to improve the speed of training. The information is transmitted forward layer by layer, starting from the input layer, passing through the hidden layer to the output layer. The actual output of the actor network is estimated correction force, it can be calculated as

(6) $$f_{e}=\sigma_{n}\left(\left(\cdots \sigma_{2}\left(\sigma_{1}\left(W\theta_{m1}\right)\theta_{m2}\right)\cdots \right)\theta_{mn}\right)$$

where $f_{e}$ is the estimated correction force, $\left(\sigma_{1,}\sigma_{2},\sigma_{3}\cdots \sigma_{n}\right)$ are activation functions for each layer, $\left(\theta_{m1,}\theta_{m2},\theta_{m3}\cdots \theta_{mn}\right)$ are the network parameters for each layer, W is the deformation of the mirror.

##### Backward Propagation Phase

The output of the actor network is divided into two types: actual output and ideal output. $f_{e}$ is the actual output, and the ideal output was f. Therefore, the loss function can be obtained as

(7) $$L\left(\theta_{m}\right)=\frac{1}{n}\overset{n}{\underset{i=1}{\sum }}{\left(f_{i}-f_{ei}\right)}^{2}$$

where $\theta_{m}$ is the network parameters for each layer, i is the node order of the output layer, n is the number of nodes in the output layer, $L\left(\theta_{m}\right)$ is the mean square error of f and $f_{e}$, and its function is to adjust the parameters of the actor network to make the prediction more accurate. In the backward propagation phase, the optimizer is needed to optimize the loss function. An optimization algorithm based on stochastic gradient descent called Adam [28], which can automatically adjust the learning rate according to the value of the gradient and improve training efficiency.

#### 2.2.3. The Strategy Unit

The output of the actor network is the estimated force, which is not optimal. Therefore, it is necessary to improve the accuracy of the correction force. When the estimated force is received, the random correction force is added in its field to obtain the correction force in all directions as much as possible. Then the correction force is input into the active optics system respectively and the value of RMS can be detected by the interferometer. Finally, the search will be stopped when the requirements are met. However, this aimless search method occupies significant computing resources and takes a lot of time.

Therefore, the heuristic search algorithm is introduced to reduce the search time. The heuristic search is a kind of method based on the estimated force output by the actor network, but it will further explore and find the optimal correction force. The heuristic search uses the existing information related to the problem as a basis to improve search efficiency and reduce the search times. The heuristic function can be defined as

(8) f(n)=g(n)+h(n)

where f(n) is the comprehensive priority of node n, g(n) is the cost of node n from the starting point, and h(n) is the cost of node n from the endpoint. When the algorithm is to jump to the next node to be searched, the direction that can make f(n) be the largest is always selected.

**Figure 4:**
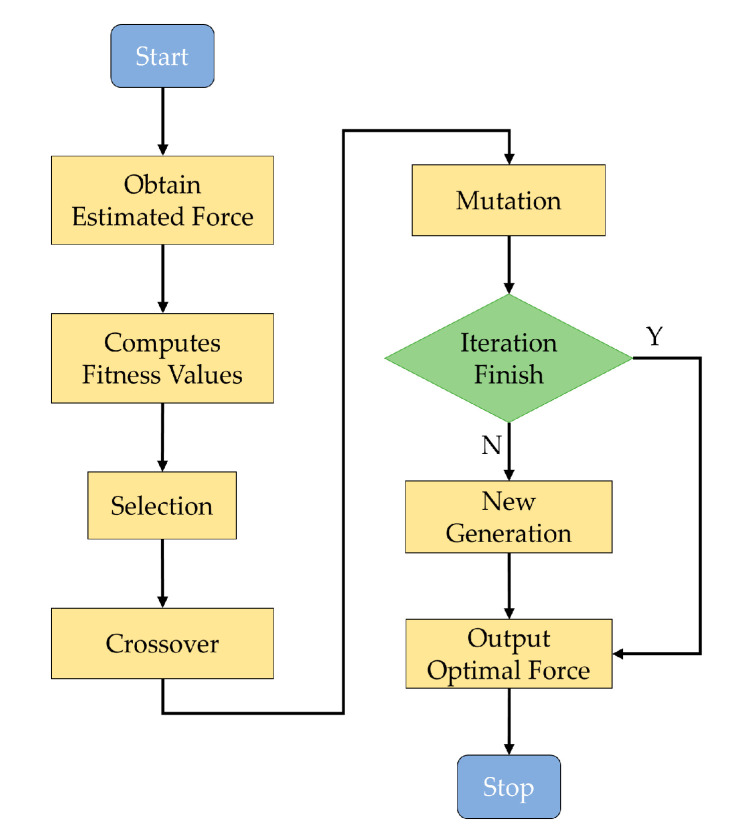
Flow chart for the correction force search with evolutionary strategy algorithm.

The heuristic search includes many mature algorithms, such as the stochastic parallel gradient descent (SPGD) algorithm [29], the simulated annealing algorithm [30,31], the ant colony algorithm [32], the hill-climbing algorithm [33], the genetic algorithm [34], the greedy algorithm [35], and the evolutionary strategy algorithm [36,37,38]. The evolutionary strategy algorithm is used to accelerate the search in this study due to the excellent performance of parallel computing. The main optimization processes include initialization, selection, mutation, crossover, and evolution, as shown in Figure 4.

#### 2.2.4. The Working Procedure of the DLCA

**Figure 5:**
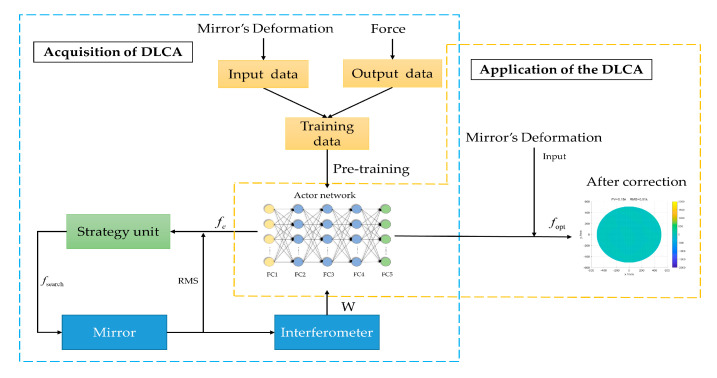
The working procedure of the DLCA.

The working procedure of the DLCA is illustrated in Figure 5, and it includes four parts: (1) Generation of datasets: To validate the feasibility of the DLCA, the training data is required to establish the mapping of the active optics system with the disturbance. Based on the analysis of Section 2.1.2, the training data can be obtained efficiently. (2) The pre-training of the actor network: The actor network is trained in advance with the training data containing the disturbance. Then, the loss function of the network is established according to the actual output and the ideal output. The gradient descent algorithm is used to minimize the loss function and update parameters. (3) Correction force optimization: The strategy unit is utilized to optimize the correction force, and all possible strategies are input into the active optics system for validation. When the optimal correction force is obtained, the input of the actor network will be updated. (4) Application of the DLCA: The well-trained actor network is used to determine the correction force.

## 3. Results 

### 3.1. Determination of Network Parameters and Datasets Update

#### 3.1.1. The Parameters Setting of the Actor Network

To construct and test the actor network, the 5000 collected data were segregated into a training set, a validation set, and a testing set in a manner consistent with a 3:1:1 ratio [39]. All three sets of data have different roles. The training set is used to identify parameters and constructs the actor network, the validation set is applied to evaluate the actor network, and the testing set is utilized to judge the final performance of the actor network. Detailed parameter settings of the actor network are listed in Table 2.

#### 3.1.2. The Configuration of the Strategy Unit

To improve the correction accuracy of the active optics system, the evolutionary strategy algorithm mentioned above was applied to reduce the search time. The 21 correction forces were output by the actor network with precision to two decimal places and reserve as the chromosomes. Then the range of actuator output was defined as −20.00 N < f < 20.00 N. Finally, the merits and demerits of individuals were evaluated by RMS value. Because this study is based on simulation, the RMS value can be obtained by MATLAB.

**Figure 6:**
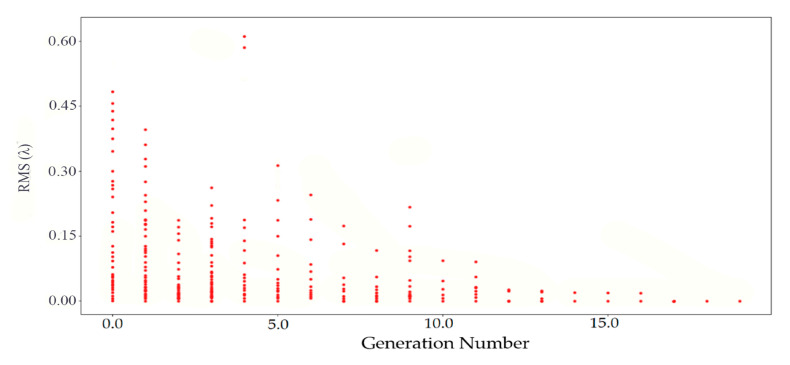
The search process of the strategy unit.

The searching process of the strategy unit is shown in Figure 6.

The various evaluation values of the initial generation reflect the variance of the individual. As the number of generations increases, the red points in the strategy unit gradually gather together, although some red points become irregular due to mutation. In the final stage, all red points were assembled in similar values until the algorithm converges, which means that the search is over and the optimal force has been found.

#### 3.1.3. Datasets Update

To further improve the correction accuracy of the proposed algorithm, the datasets were updated circularly. In the beginning, the parameters of the actor network and the strategy unit were randomly initialized. The actor network was trained in advance according to the datasets obtained by MATLAB. After training, the actor network output the estimated force according to the initial mirror shape. Then, the evolutionary strategy algorithm was used in the strategy unit, which makes a pass through all of the available individuals in every iteration and predicts the RMS value of each individual. It will kill the weaker individuals until all of them converge to one place. Finally, the optimal correction force was applied to the mirror. The corrected mirror shape was judged whether it can meet the requirements, if not, the correction process will be executed again; otherwise, the correction will be finished. At the end of the correction, a set of random correction forces were inputted to disturb the mirror shape after correction for the next training. The above description was implemented in MATLAB. Specifically, the data during each correction, including the mirror shape before correction, the optimal correction force, and the mirror shape after correction, were updated to the datasets.

### 3.2. Comparison of Different Correction Algorithm

In this study, a random disturbance, which follows the normal distribution of μ = 0 and σ = 1, was added to the stiffness matrix to simulate the disturbances in the real system by MATLAB. To validate the correction capability and feasibility of the proposed algorithm, five different types of initial mirror shapes, including RMS of 0.26λ, 0.44λ, 0.68λ, 0.84λ, and 1.07λ, were selected. Simultaneously, the most widely used least square algorithm was chosen as a comparison to carrying out the correction force under the same condition, the specific calculation of the least square algorithm refers to [11]. 

**Figure 7:**
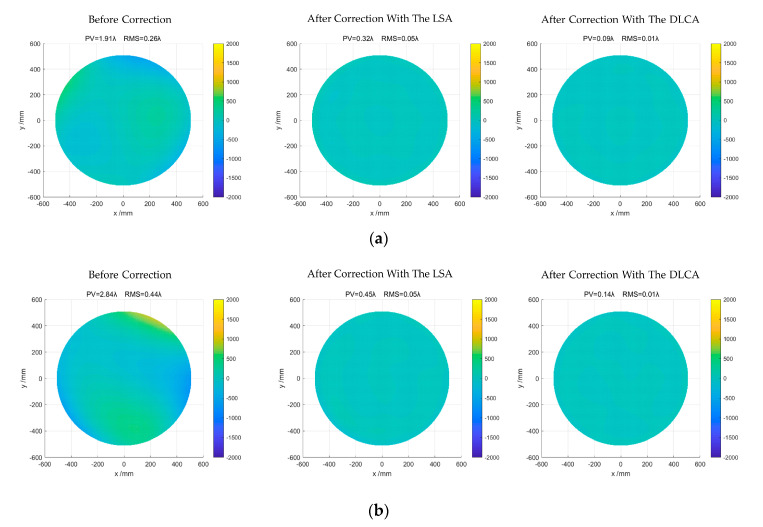

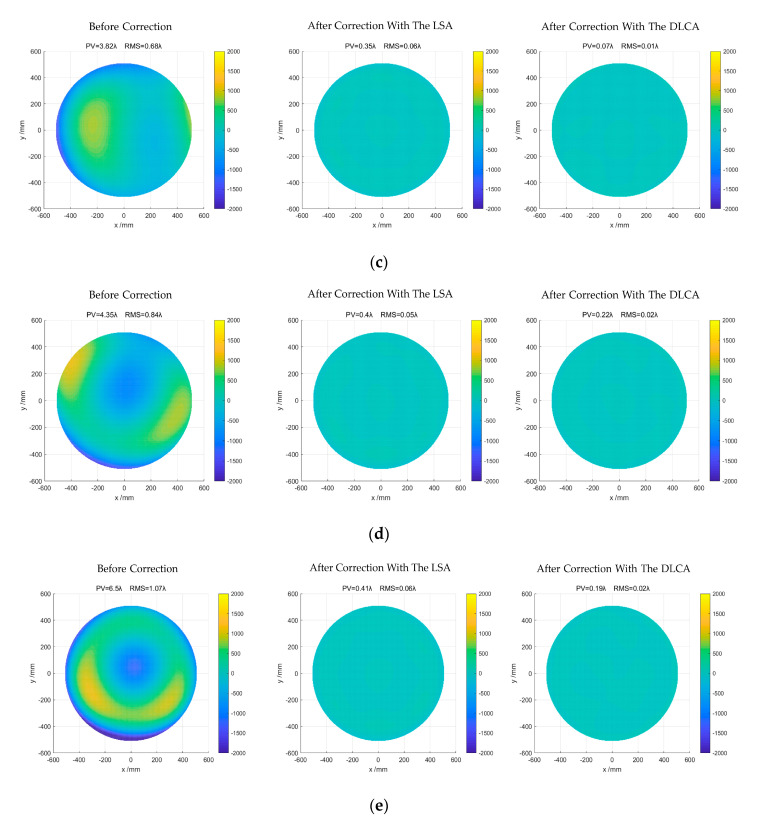
Mirror shape before and after correction. (**a**) The initial RMS (Root Mean Square) of the mirror is 0.26λ; (**b**) The initial RMS of the mirror is 0.44λ; (**c**) The initial RMS of the mirror is 0.68λ; (**d**) The initial RMS of the mirror is 0.84λ; (**e**) The initial RMS of the mirror is 1.07λ.

Figure 7 shows the correction results of the least square algorithm (LSA) and the DLCA. From left to right are the initial mirror shape before correction, the mirror shape is corrected by the LSA, and the mirror shape is corrected by the DLCA.

Apparently, the deformation of the mirror was modified by the two algorithms after correction. Notably, the correction performance of the DLCA is always better than the LSA regardless of the initial mirror shape. The detailed correction results of the LSA and the DLCA are shown in Table 3.

In the first experiment, the initial RMS of the mirror was 0.26λ. After correction with the LSA, the RMS of the mirror was reduced to 0.05λ, and the number of corrections was three. Compared with LSA, the DLCA reduces the RMS from 0.26λ to 0.01λ, which correction times are one. In the second experiment, the initial RMS of the mirror was 0.44λ. After correction with the LSA, the RMS of the mirror was reduced to 0.05λ, and the number of corrections is four. Compared with LSA, the DLCA reduces the RMS from 0.44λ to 0.01λ, which correction times are still one. In the third experiment, the initial RMS of the mirror was 0.68λ. After correction with the LSA, the RMS of the mirror was reduced to 0.06λ, and the number of corrections was still four. Compared with LSA, the DLCA reduced the RMS from 0.68λ to 0.01λ, whose correction times are one.

To further illustrate the feasibility of the proposed algorithm, the initial RMS of the mirror was set worse. Therefore, in the fourth and fifth experiments, the initial RMS of the mirror was 0.84λ, and 1.07λ. After correction with the LSA, the RMS of the mirror was reduced to 0.05λ and 0.06λ. However, the number of corrections increased to 5 and 6. Compared with LSA, the DLCA reduced the RMS from 0.84λ to 0.02λ and 1.07λ to 0.02λ. The correction times were all two. 

According to the above results, we can find that the LSA takes about 3–5 times on average to complete the correction, and the RMS value after the correction is about 0.05λ. While the DLCA only needs 1–2 times to complete the correction, and the RMS value after the correction is about 0.01λ. Therefore, we can conclude that DLCA has better correction performance and can effectively reduce the RMS of the mirror. Compared with LSA, DLCA only needs fewer correction times and has higher correction accuracy. 

**Figure 8:**
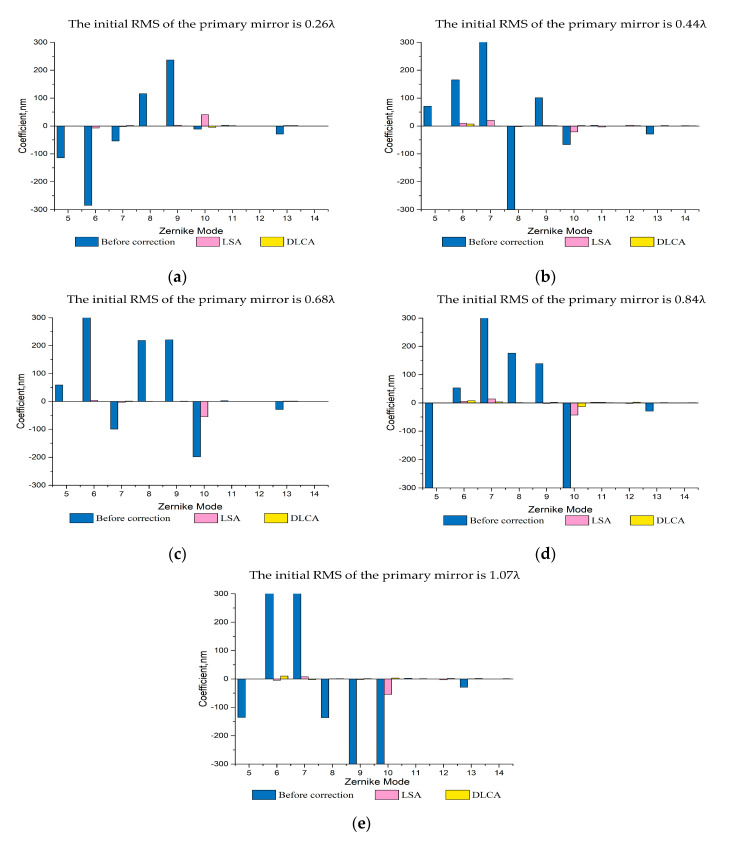
The correction results of LSA and DLCA in Zernike mode. (**a**) The initial RMS of the mirror is 0.26λ; (**b**) The initial RMS of the mirror is 0.44λ; (**c**) The initial RMS of the mirror is 0.68λ; (**d**) The initial RMS of the mirror is 0.84λ; (**e**) The initial RMS of the mirror is 1.07λ.

Furthermore, to illustrate the superiority of the proposed algorithms, we compared the correction performance of the LSA and DLCA on Zernike mode. Since the four items of Zernike polynomial, translation, tilt, and defocus, can be corrected by other methods, the four items were not selected. Meanwhile, considering the poor ability of active optics to correct higher-order aberrations, only the 5th–14th items are selected. As mentioned above, five types of initial mirror shapes were also selected, the correction results of the LSA and the DLCA in Zernike mode are shown in Figure 8.

As can be seen from Figure 8, compared with the LSA, the low order Zernike aberrations, especially the astigmatisms, were significantly reduced after correction when adopting the DLCA. Additionally, the reduction of high order Zernike aberrations is relatively weak, which shows that the correction capability for high order Zernike aberrations is poorer than the low order Zernike aberrations. Based on the analysis above, we can find that the DLCA has a stronger ability to correct aberrations than LSA. 

## 4. Discussion

The support system of the active optics platform consists of 24 axial supports and 3 lateral supports in the experiments. Under this condition, the proposed algorithm has excellent correction performance. However, this does not affect the application of the proposed algorithm in other conditions, nor does it affect the universality of the proposed algorithm. In other words, regardless of the number and distribution of actuators, the proposed algorithm in this study can also calculate the correction force.

To simulate the real system, a random disturbance is generated and added to the stiffness matrix by MATLAB in this paper. Therefore, all the datasets used for training contain the disturbance. For the disturbance of other factors, the algorithm proposed in this paper can also be applied, only if the corresponding disturbance is added to the datasets. In other words, the datasets used for training must contain the corresponding disturbance, so that the deep neural network can fit the active optics system with disturbances. For the real system, this is easier to implement because of the input and output data collected by the real system that already contains various disturbances, such as thermal distortion, and gravity deformation, especially the error in structure and actuator control.

In addition, to adequately illustrate the advantages of the proposed algorithm in correction performance, the comparison of the least square algorithm and the algorithm proposed in this paper is carried out. The data of Table 3 shows that the correction performance of the proposed algorithm is always better than the LSA regardless of the initial mirror shape, which indicates that the proposed algorithm has a better correction capability that deals with the mirror shape with the disturbance. Therefore, we conclude that the proposed algorithm has better adaptability and correction capability than the traditional algorithm in the case of the disturbance.

From the above results, it is known that the proposed algorithm can correct wavefront aberrations more effectively than the traditional algorithm. However, there are still many high-order aberrations that cannot be corrected, which indicates that the proposed algorithm has a good correction performance of the low-order aberrations, but the ability to correct high-order aberrations is relatively weak. Although the mirror shape may have been corrected well, there are still high-order aberrations. In this case, only adaptive optics can be used for correction with its advantage of correcting the high-order aberrations. 

## 5. Conclusions

A deep learning correction algorithm is proposed in this paper for the correction of wavefront aberrations, which is based on the data rather than the mirror’s deformation. Therefore, the proposed algorithm can deal with the active optics system with disturbances better. Five different types of initial mirror shapes, including 0.26λ, 0.44λ, 0.68λ, 0.84λ, and 1.07λ, are utilized to validate the proposed algorithm. The RMS of mirror shape is significantly reduced and the low-order aberrations are also reduced after correction. Additionally, compared with the least squares algorithm, the performance of correct wavefront aberrations is overall improved, which indicates that the algorithm has a better correction capability.

The results also provide us with some inspirations for future work. Firstly, the evolutionary strategy algorithm is utilized to reduce the search time, but the search process still takes much time, especially when the number of actuators further increases. We will continue to focus on how to reduce the search time of the strategy unit. Secondly, to improve the speed of practical application, we will continue to try to design a new input way so that the correction force can be calculated directly from the figure of the mirror shape. Thirdly, we will try to apply the proposed algorithm to large modern telescopes in the future study.

The contributions of this paper are as follows: (1) A new correction algorithm based on deep learning is proposed, which can improve the correction capability of the mirror; (2) Deep learning is introduced into the active optics system and provides a new train of thought for the further research of how to improve the correction capability of the mirror. 

## Figures and Tables

**Table 1:** The key components of the standard mirror.

Name	Parameter
Diameter	1000 mm
Thickness	80 mm
Radius of Curvature	4000 mm
Material	K9 Glass
Mass	174.445 kg

**Table 2:** The parameter setting of the actor network.

FC1 Nodes	FC2 Nodes	FC3 Nodes	FC4 Nodes	FC5 Nodes	Activation Functions	Optimizer	Learning Rate
65	145	200	120	21	ReLU	Adam	0.001

**Table 3:** Comparison of correction results for different algorithms.

Method.	Before Correction	After Correction	Correction Times
LSA	0.26	0.05	3
DLCA	0.26	0.01	1
LSA	0.44	0.05	4
DLCA	0.44	0.01	1
LSA	0.68	0.06	4
DLCA	0.68	0.01	1
LSA	0.84	0.05	5
DLCA	0.84	0.02	2
LSA	1.07	0.06	6
DLCA	1.07	0.02	2
